# Methodology to analyse small silicon samples by glow discharge mass spectrometry using a thin wafer mask

**DOI:** 10.1016/j.mex.2015.10.005

**Published:** 2015-10-19

**Authors:** C. Modanese, L. Arnberg, M. Di Sabatino

**Affiliations:** Norwegian University of Science and Technology (NTNU), Department of Materials Science and Engineering, 7491 Trondheim, Norway

**Keywords:** Glow discharge mass spectrometry, Small solid samples, Thin mask, Silicon mask, Tantalum mask, Flat wafer mask, Cathode–anode distance, Bulk analysis by glow discharge mass spectrometry

## Abstract

Glow discharge mass spectrometry (GDMS) is widely used for trace element analysis of bulk solid samples. The geometry of the GD source limits the minimum size of the sample, which for the instrument used in this work (ThermoElementGD) is 20 mm in diameter. From time to time, there is the need to analyse smaller samples with this technique, and we present here a methodology to analyse samples of 9–20 mm diameter through the use of thin masks.

Thin masks have been previously used mostly as secondary cathode for the analysis of non-conducting materials, with hole size smaller than the area of the glow discharge. The use of masks in this work includes the following customization:

• The choice of highly-pure Si as mask material, to decrease the chance of interferences with the Si samples.
• The use of a hole in the mask of the same size as the discharge area. This implies that the mask material is not sputtered, thus decreasing chances for contamination from the mask itself.

## Graphical abstract

**Figure:**
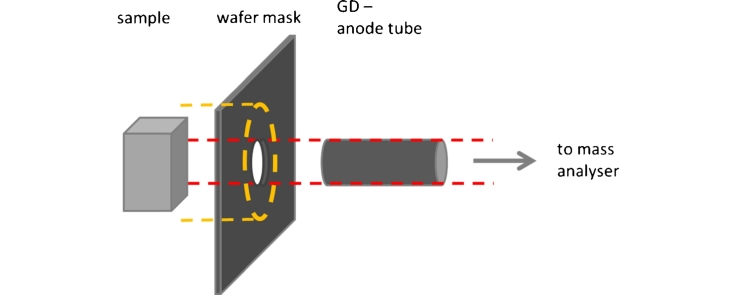

## Method details

### Methodology to analyse silicon samples by glow discharge mass spectrometry

**Fig. 1:**
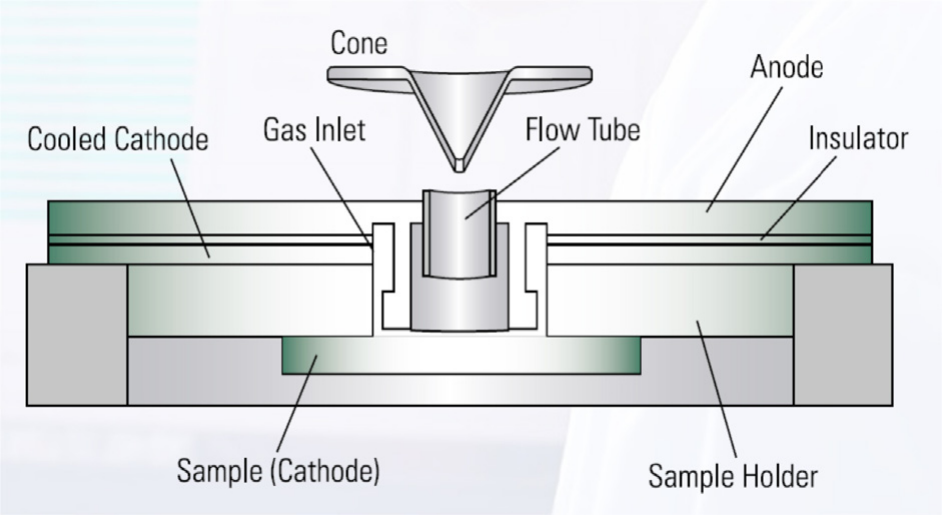
Schematic of the sample (cathode) and anode in a glow discharge.

Reprinted from [2].

Elemental analyses of silicon by direct current (dc) GDMS are generally carried out on bulk samples, which act as the cathode (see Fig. 1). The sample size is bigger than the size of the ceramic insulator ring around the anode flow tube [1], i.e. with diameter ≥20 mm for the design of the instrument used in this work. Hence, samples for routine analysis, i.e. without any mask, should have a diameter 20–70 mm and height smaller than 40 mm. The sputtered area has a diameter of 8 mm. Smaller samples may be of interest for analysis, e.g. due to the lab-scale size of the material's production process. Few alternatives, such as using a smaller anode diameter, have been suggested to address this issue. In this study we have used thin sheet masks to be positioned between the anode and the sample surface, thus allowing the vacuum to be reached.

**Fig. 2:**
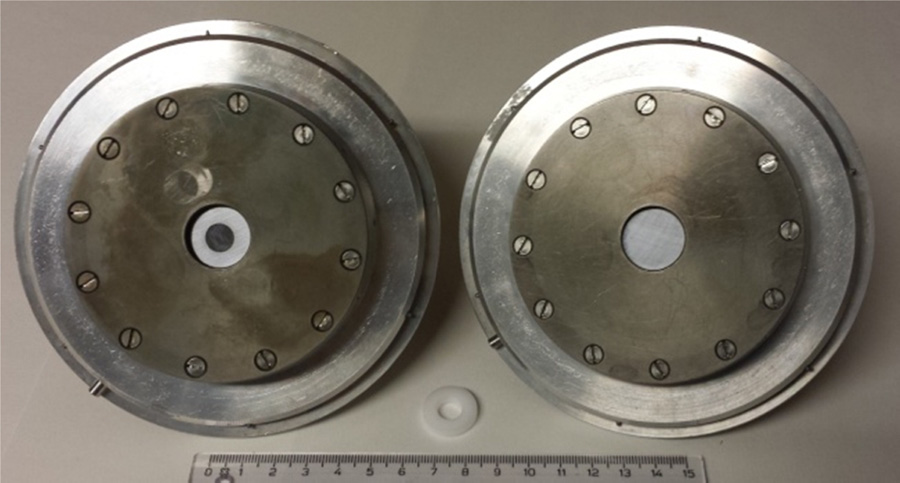
Silicon samples mounted on the sample holders, with and without mask (left and right, respectively). The centring tool (plastic ring) to position the mask prior to analysis is shown in the middle.

The thickness of the mask has an impact on the distance between the anode and the sample surface (cathode), being ∼200 μm at the beginning of the discharge. Hence, different thicknesses between 145 and 300 μm were tested. Moreover, due to the low concentration of impurities in silicon for photovoltaics (materials with purity from 6 N to 9 N), the choice of the mask material is critical to avoid external contamination and memory effects. In this study, we have tested two different materials, i.e. silicon and tantalum, to be used as masks, and analysed the Si materials with similar discharge parameters used for standard Si analyses [3], i.e. without any mask. The centring of the masks on the anode was done manually, with the aid of a custom-made plastic tool to assist in the final adjustments. After the mask was centred and the Si sample correctly positioned, the sample holder was fastened as usually and mounted on its support. Fig. 2 shows two sample holders with and without the mask as well as the centring tool (plastic ring).

### Preparation of thin silicon wafer masks

Silicon wafers for photovoltaic applications were used as masks. The wafers were from two different materials and with two different thicknesses, i.e. mono- and multicrystalline Si wafers with thickness of 145 μm and 300 μm, respectively.

The wafer masks were prepared as follows:

1. A 30 mm × 30 mm square mask was laser cut from the original wafer.
2. A hole was laser cut in the middle of the mask, with size 6, 8 or 10 mm. Note that 8 mm is the diameter of the flow tube in the anode.
3. In order to avoid contamination from the laser cutting onto the internal ring in the mask, each mask was etched with the following procedure:
  I. Removal of organic residues by cleaning in RCA1 solution (ratio 5:1:1 – H_2_O:NH_3_:H_2_O_2_), 10 min.
  II. Dipping in 5% HF, 3 min.
  III. Removal of metal ions and inorganic residues by cleaning in RCA2 solution (ratio 5:1:1 – H_2_O:HCl:H_2_O_2_), 10 min.
4. After etching, the mask was stored in a clean box and handled only with gloves.
5. The roughness of the wafers has been calculated as:

$$\text{roughness}=\sum_{i}\frac{\left(y_{i+1}-y_{i}\right)}{l}$$

where *y*_*i*_ is the sum of the height differences between neighbour points on the surface and *l* is the total measured length. The roughness was measured along a 5 mm line with a step resolution of 12 μm and it is 0.11 and 0.16 for the 145 μm and 300 μm thick wafers, respectively. The planarity of the wafers is excellent for the application purpose.

### Preparation of thin tantalum sheet masks

High-purity tantalum sheets (Ta–W alloy) provided by H.C. Starck were used as masks. The thickness of the sheets is 140–170 μm, i.e. the sheets are not completely flat and present bending.

The masks were prepared as follows:

1. A 25 mm × 25 mm square mask was laser cut from the original sheet.
2. A hole was laser cut in the middle of the mask, with size 8 mm. Note that this is the diameter of the flow tube in the anode.
3. In order to avoid contamination from the laser cutting onto the internal ring in the mask and from the production process in general, each mask was etched with the following procedure:
  I. Cleaning in chromic acid solution (0.1 mol/l K_2_Cr_2_O_7_ dissolved in H_2_SO_4_), 10 min (or until needed), at room temperature.
  II. Rinsing with abundant DI water.
4. The inner side of the laser cut hole was drilled with a clean diamond tip in order to smooth the edge shape.
5. The mask was stored in a clean box and handled only with gloves.

The roughness of the sheets was measured to be 0.5 using the same procedure for the Si wafers.

The as-received Ta sheets are not as flat as the silicon wafers, i.e. they present curvature of the surface. This implies that the physical contact between the flat surface of the Si sample and the slightly curved surface of the Ta mask may lead to a small gap. In turn, this gap will lead to a partial discharge below the surface of the mask, i.e. between the mask and the sample surface. Although the surface roughness of the Ta sheets was measured to be higher than that of the Si wafers, its effect is believed to be of second order compared to the effect of the curvature of the sheets. A procedure to flatten the Ta masks was tried with limited success, and more details are reported on Appendix B. The results of the analysis after the flattening are reported alongside the ones obtained on the as-received masks.

### Comparison between the use of Si or Ta as mask materials

Silicon and tantalum present both advantages and disadvantages related to their use (Table 1).

The analyses show that the use of the thin Si mask (145 μm) and the 8 mm hole diameter (i.e. corresponding to the inner diameter of the anode) does not introduce significant variations in the measured concentrations, and may therefore be used for analyses of small samples.

### Analytical parameters

In order to evaluate the error introduced by the use of the masks, the analyses were compared with analyses on the same Si samples without any mask. The discharge parameters used to analyse the silicon samples with the use of masks are similar to the parameters used for analysis without any mask, i.e. standard parameters. This allows using the same correction factors for quantification in the different analyses. The parameters are listed in Table 2. No significant changes in the pumping time were observed.

### Method validation

**Fig. 3:**
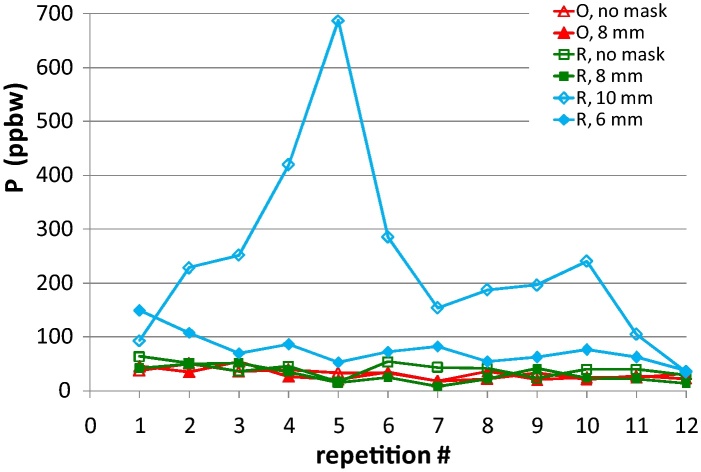
Concentration of phosphorus over repetition #, analysed with 6, 8 and 10 mm hole diameter and without any mask. It can be seen that the instabilities are greater for the 6 and 10 mm masks.

The following aspects were observed during analysis with the Si masks:

• With the thinner masks (145 μm), the discharge voltage is generally lower than without any mask, and occasionally there are random bounces of up to ±40 V lasting 1–2 s. These bounces were not observed to have a marked effect on the ion beam ratio, thus on quantified concentration of the impurities.
• With the thicker masks (300 μm), the discharge voltage is showing greater bounces compared to the thinner mask, up to ±60 V for 1–2 s. The peaks of the isotopes are generally observed to have a slight mass shift compared to analyses with the thinner mask or without any mask. The background is higher than usual, especially for the ^11^B and ^31^P isotopes. The sample temperature after analysis is sensibly higher to the touch, and the mask is sticking onto the sample surface until cooling down to room temperature. However the temperature during analysis was not monitored.
• With either the largest hole (10 mm Ø) or the smallest hole (6 mm Ø), the discharge voltage is sensibly lower, and several isotope peaks present one or two high spikes, not homogeneously distributed over the mass range and over repeated analyses in the same crater. Furthermore, the background noise with the 6 mm Ø mask is sensibly higher, even hiding the real isotope peak in some repetitions. This was observed for all the impurity isotopes, at different sputtering times, and it appears to occur randomly. The measured concentrations of phosphorus are reported in Fig. 3 as an example of the instabilities in the signal during the analysis with the 6 mm and 10 mm hole diameter masks compared to analysis with the 8 mm hole diameter mask or without the mask.

The following aspects were observed during analysis with the Ta masks:

• The ion beam signal is frequently lost, either permanently or for a few minutes before returning back to former values. This may be related to the presence of a small gap between the Ta mask and the Si sample due to the curvature of the Ta sheet, which leads to an unstable physical contact and hence an unstable discharge. A signal loss was observed in approximately 50% of the attempted analyses and it was observed to be connected to higher impurities concentrations.
• Due to the poor flatness of the Ta sheets, the crater depth was observed to be non-symmetrical, with a higher sputtering rate on the side possibly corresponding to a gap between the Ta mask and the Si sample. The difference in the sputtering rate is rather marked, being more than 1.5 times the usual value.
• The contamination from the Ta mask appears to be more relevant with a second use of the mask. However, it is not clear whether it is due to a different positioning of the mask on the sample holder or to a contamination of the inner ring of the mask from the previous analyses. The stability of the Ta signal between repeated analyses with the same mask seems to show that the latter is more probable. Since the main advantage of the use of the Ta masks over the Si masks is their reusability for consecutive analyses, etching of the masks before the second use has not been considered in this work.
• The intensity (ion beam ratio) of the Ta signal is very different among different masks, e.g. up to 3 orders of magnitude.

In order to validate the use of the masks for bulk analysis, the results of few analyses are reported in Table 3. Both selected Si and Ta masks are reported, and compared to analyses on the same silicon samples without any mask, i.e. the standard analytical layout for Si analysis by GDMS. The material reported here, *R*, is representative of standard multicrystalline silicon materials for photovoltaics, i.e. 9 N purity. The concentration of all impurities except Ta are fully quantitative, and are calculated according to the calibration factor, i.e. relative sensitivity factors (RSF), calculated for each analyte according to the procedure reported in Ref. [4]. The RSF for Ta is not available, and therefore the Ta analyses are reported as ion beam ratio (IBR), i.e. semi-quantitative. More complete data, including further measurements and all the repetitions per crater (i.e. where the initial 10 min of sputter are included), are reported in Appendix A. The analyses also show that B could be measured with both masks and also with the mask being re-used (i.e. side 2 analysis). However, the remaining elements show low reproducibility of the measured concentrations and generally greater error, as indicated in Appendix A. Hence the use of the Ta masks appears to be suitable only for B analysis, among the analysed impurities.

## Figures and Tables

**Table 1:** Advantages and disadvantages related to the use of Si and Ta as materials for the masks.

	Advantages	Disadvantages
Si	Highly pure materials (9 N) are available. Thus the risk of contamination is negligible. Processing and cleaning of the silicon wafers is well known and fast. Masks are perfectly planar.	Single use. (Due to brittle behaviour at RT and the thermal/mechanical stress undergone during analysis, the probability of fracture of the Si mask after the first analysis was observed to be ≫50%.)
Ta	Repeated use may be possible. Relatively pure material, with low concentrations (∼ppm) of impurities relevant for Si applications.	Poor planarity. (Difficult to obtain due to the mechanical properties of Ta. This impacts the geometry of the GD at the sample surface.)

**Table 2:** Discharge parameters used for the analysis of silicon samples with the use of Si or Ta thin masks between cathode (sample) and anode.

Discharge voltage	630–870 V
Discharge current	57.6 mA
Discharge gas	395 mL/min
Matrix signal intensity (^28^Si)	10^9^–10^10^ cps
Total sputtered depth	20–25 μm

**Table 3:** Concentration of impurities measured with and without Si or Ta mask on sample R. All elements except Ta are fully quantified, whereas Ta is shown as ion beam ratio, IBR, i.e. semi-quantitative. Ta #1 and Ta #2 refer to different masks, whose thickness is 140–170 μm.

Mask type and hole Ø	Si matrix (cps)	Concentration (ppbw)					IBR (ppbw)
		B	Al	P	Fe	Cu	Ta
None	1.7 × 10^9^ ± 2 × 10^7^	115 ± 14	33 ± 13	39 ± 10	2.0 ± 1.7	169 ± 59	NA
Si, 145 μm, 8 mm	1.9 × 10^9^ ± 4 × 10^7^	108 ± 8	55 ± 30	22 ± 11	6.1 ± 3.4	144 ± 43	NA
Si, 300 μm, 8 mm	1.6 × 10^9^ ± 3 × 10^7^	127 ± 14	9555 ± 2619	23 ± 14	22.3 ± 5.3	203 ± 51	NA
Si, 145 μm-side1, 10 mm	4.6 × 10^9^ ± 5 × 10^7^	257 ± 69	129 ± 48	172 ± 83	71 ± 40	438 ± 116	NA
Si, 145 μm, 6 mm	4.5 × 10^9^ ± 9 × 10^7^	2058 ± 4939	703 ± 1458	179 ± 232	47 ± 65	503 ± 180	NA
Ta #1, 8 mm	7.6 × 10^9^ ± 2 × 10^8^	108 ± 8	31 ± 7	13 ± 10	4.2 ± 5.8	7.6 ± 13.3	4.1 × 10^4^ ± 2.8 × 10^4^
Ta #2, side1, 8 mm	1.5 × 10^10^ ± 6 × 10^8^	119 ± 6	108 ± 103	20 ± 10	42 ± 23	939 ± 777	2.3 × 10^7^ ± 2.3 × 10^6^
