# Supplementary material for: Methodology to analyse small silicon samples by glow discharge mass spectrometry using a thin wafer mask
Source: MethodsX. 2015 Oct 19;2:409–14. doi: 10.1016/j.mex.2015.10.005 (PMC4644257; doi:10.1016/j.mex.2015.10.005)
Supplement: Supplementary file 1 [file mmc1.pdf]

Appendix A. Supplementary data

Glow discharge mass spectrometry (GDMS) [A1]

GDMS is an analytical technique based on a glow discharge ion source that generates ions through sputtering of a solid surface. The GD plasma is created when an electric current passes through a low-pressure gas, and a differential potential is established between the cathode (sample) and the anode. The ions generated in the discharge are separated and detected in a mass spectrometer.

GDMS is a method widely used for determination of trace elements in solid samples, both in conductive and non-conductive materials. A direct-current (dc) operated GD source can analyse only conductive materials, whereas a radio frequency (rf) source can analyse both conductive and non-conductive materials.

Note that the instrument used in this work is a high resolution dc GDMS (ThermoElementGD).

[A1] Venzago and Pisonero, New Developments in Mass Spectrometry No. 3, chapter 13, Ed. Prohaska, Irrgeher, Zitek, Jakubowski, The Royal Society of Chemistry, 2015, p. 319-378

Analytical results with thin silicon wafer masks

Average and standard deviation of the analyses carried out with Si wafer masks of different thickness, i.e. 145 and 300 µm, and with different hole diameters, i.e. 6, 8 and 10 mm, are reported below. The values are calculated including all repetitions on the same crater (Table A1), i.e. with only 2 min of pre-sputter, and including only the repetitions after 10 min of (pre-)sputter (Table A2), i.e. as usual for bulk analysis. The two materials reported here are R and O, both multicrystalline Si samples of 9 N purity.

Table A1. Average and standard deviation of the concentrations measured with the Si masks, all repetitions on the crater included, i.e. only 2 min pre-sputter time.

Sample	Mask type and hole Ø	Si matrix (cps)	Concentration (ppbw)				
			B	Al	P	Fe	Cu
O	None	$2.9 \times 10^9 \pm 1 \times 10^8$	71±10	26±5	31±9	2.6±2.7	114±22
O	145µm, 8mm	$2.9 \times 10^9 \pm 4 \times 10^7$	76±12	78±78	31±11	7.0±6.6	95±19
O	300µm, 8mm	$1.6 \times 10^9 \pm 5 \times 10^7$	491±807	17737±6341	285±308	257±418	1447±687
R	None	$1.6 \times 10^9 \pm 5 \times 10^7$	119±13	41±15	40±13	2.8±2.0	191±55
R	145µm, 8mm	$1.9 \times 10^9 \pm 4 \times 10^7$	103±10	107±74	29±14	14.5±20.4	163±52
R	300µm, 8mm	$1.6 \times 10^9 \pm 3 \times 10^8$	126±23	12951±8226	30±16	26.3±12.7	261±97
R	None	$8.4 \times 10^9 \pm 5 \times 10^7$	107±6	53±47	46±6	77±181	628±172
R	145µm-side1, 10mm	$4.6 \times 10^9 \pm 8 \times 10^7$	311±142	162±68	240±173	117±85	651±338
R	145µm-side2, 10mm	$5.3 \times 10^9 \pm 8 \times 10^7$	151±10	52±29	29±6	9.9±8.4	180±28
R	145µm, 6mm	$4.6 \times 10^9 \pm 8 \times 10^7$	2067±4484	1358±2128	185±192	227±612	2506±6981

Table A2. Average and standard deviation of the concentrations measured with the Si masks, only the repetitions after 10 min sputter included.

Sample	Mask type and hole Ø	Si matrix (cps)	Concentration (ppbw)				
			B	Al	P	Fe	Cu
O	None	$3.0 \times 10^9 \pm 1 \times 10^8$	70±11	24±4	25±6	1.7±3.2	100±14
O	145µm, 8mm	$2.9 \times 10^9 \pm 3 \times 10^7$	79±13	45±9	27±7	3.9±2.7	85±14
O	300µm, 8mm	$1.6 \times 10^9 \pm 2 \times 10^7$	735±1013	13164 ±3517	364±380	355±541	1550±775
R	None	$1.7 \times 10^9 \pm 2 \times 10^7$	115±14	33±13	39±10	2.0±1.7	169±59
R	145µm, 8mm	$1.9 \times 10^9 \pm 4 \times 10^7$	108±8	55±30	22±11	6.1±3.4	144±43
R	300µm, 8mm	$1.6 \times 10^9 \pm 3 \times 10^7$	127±14	9555±2619	23±14	22.3±5.3	203±51
R	None	$8.4 \times 10^9 \pm 4 \times 10^7$	109±6	36±5	33±6	11.0±1.3	524±50
R	145µm, side1, 10mm	$4.6 \times 10^9 \pm 5 \times 10^7$	257±69	129±48	172±83	71±40	438±116
R	145µm, side2, 10mm	$5.3 \times 10^9 \pm 1 \times 10^8$	151±9	49±9	26±5	10.0±9.6	177±27
R	145µm, 6mm	$4.5 \times 10^9 \pm 9 \times 10^7$	2058±4939	703±1458	179±232	47±65	503±180

Analytical results with thin tantalum sheet masks

Average and standard deviation of the analyses carried out with two different Ta masks, i.e. #1 and #2, are reported below. The values are calculated including all repetitions on the same crater (Table A3), i.e. with only 2 min of pre-sputter, and including only the repetitions after 10 min of (pre-)sputter (Table A4), i.e. as usual for bulk analysis. Note that during the analyses with mask #2 the signal was lost; hence the reported values are averaged over a lower number of repetitions. Material R is the same used for the analyses with the Si masks. Ta masks appears to be suitable only for B analysis, among the analysed impurities.

Table A3. Average and standard deviation of the concentrations measured with the Ta masks on sample R, all repetitions on the crater included, i.e. only 2 min pre-sputter time.

Mask type and hole Ø	Si matrix (cps)	Concentration (ppbw)					IBR (ppbw)
		B	Al	P	Fe	Cu	Ta
None	$7.6 \cdot 10^9$ $\pm 3 \cdot 10^8$	122±8	35±3	16±4	3.2±6.2	28±36	$7 \cdot 10^0$ $\pm 1 \cdot 10^0$
Ta #1, 8mm	$7.6 \cdot 10^9$ $\pm 2 \cdot 10^8$	108±8	31±7	13±10	4.2±5.8	7.6±13.3	$4.1 \cdot 10^4$ $\pm 2.8 \cdot 10^4$
Ta #2, side1, 8mm	$1.5 \cdot 10^{10}$ $\pm 6 \cdot 10^8$	119±6	108±103	20±10	42±23	939±777	$2.3 \cdot 10^7$ $\pm 2.3 \cdot 10^6$
Ta #2, side2, 8mm	$1.4 \cdot 10^{10}$ $\pm 9 \cdot 10^8$	137±18	638±869	97±73	533±473	2347±1462	$2.5 \cdot 10^7$ $\pm 1.6 \cdot 10^6$

Table A4. Average and standard deviation of the concentrations measured with the Ta masks on sample R, only the repetitions after 10 min sputter included.

Mask type and hole Ø	Si matrix (cps)	Concentration (ppbw)					IBR (ppbw)
		B	Al	P	Fe	Cu	Ta
None	$7.3 \cdot 10^9$ $\pm 1 \cdot 10^8$	121±7	34±3	13±3	1.2±0.7	17±8	$7 \cdot 10^0$ $\pm 1 \cdot 10^0$
Ta #1, 8mm	$7.5 \cdot 10^9$ $\pm 2 \cdot 10^8$	106±7	29±7	10±3	1.1±0.3	2.0±2.6	$2.3 \cdot 10^4$ $\pm 1.2 \cdot 10^4$
Ta #2, side1, 8mm	$1.4 \cdot 10^{10}$ $\pm 5 \cdot 10^8$	116±4	71±26	16±2	32±4	425±130	$2.2 \cdot 10^7$ $\pm 1.8 \cdot 10^6$
Ta #2, side2, 8mm	$1.4 \cdot 10^{10}$ $\pm 6 \cdot 10^8$	125±5	228±17	63±14	2423±32	1144±224	$2.3 \cdot 10^7$ $\pm 1.3 \cdot 10^6$

The poor flatness of the Ta masks affected the symmetry of the sputtered crater, i.e. the craters were non-symmetric due to a partial discharge occurring in the thin gap between the mask and the sample surface. An example of a non-symmetric crater with the Ta mask is shown in Figure A1.

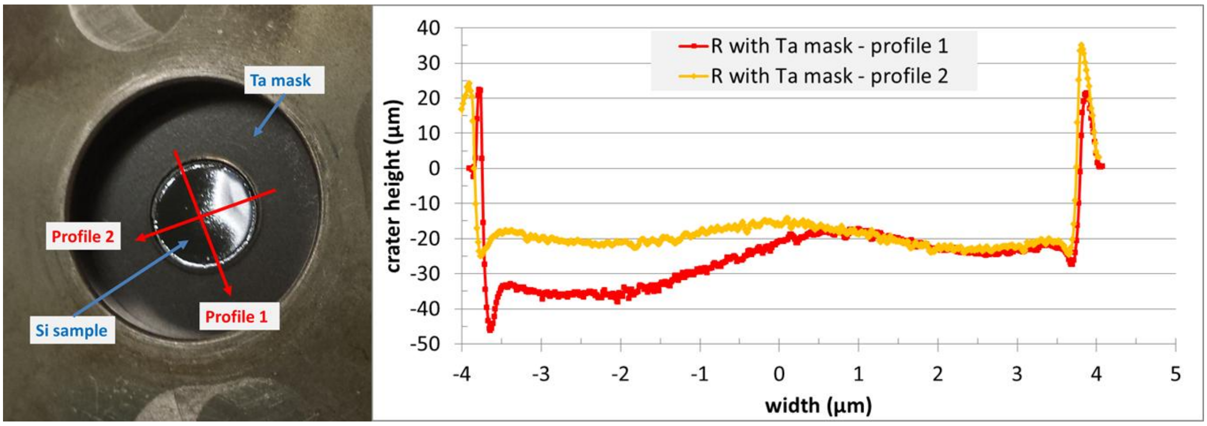

Fig. A1. Non-symmetric shape of the crater sputtered with a Ta mask. Direction of the line profile acquisition (left) and depth profile (right).
