# Supplementary material for: Methodology to analyse small silicon samples by glow discharge mass spectrometry using a thin wafer mask
Source: MethodsX. 2015 Oct 19;2:409–14. doi: 10.1016/j.mex.2015.10.005 (PMC4644257; doi:10.1016/j.mex.2015.10.005)
Supplement: Supplementary file 2 [file mmc2.pdf]

Appendix B. Additional information

In order to flatten the surface of the Ta sheets, the following procedure was tested:

1. A Ta sheet is placed between two slabs of steel. In order to allow for flattening, the steel surfaces in contact with the Ta sheet are mechanically polished down to 1 µm.
2. The steel slabs with the Ta sheet in between are put under a press, and pressed under a high-load, i.e. overcoming the yield strength of the Ta sheet. Thus the sheet should deform plastically and become flatter.
3. After plastic deformation, a hole is laser cut on the Ta sheet, thus obtaining the mask.
4. The Ta mask is then cleaned in chromic acid solution for 10 min, at room temperature.

This procedure was attempted using steel slabs that revealed to be weaker than the Ta sheet, i.e. they started deforming plastically and hence did not provide an optimal flattening for the Ta sheet. However, the thickness of the Ta sheet was measured to have smaller variations over the sheet surface compared to as-received sheets, with thickness in the range 145 and 155 µm. Note that the aim of the flattening procedure was to straighten out the curvatures in the Ta sheets rather than decreasing the variations in thickness itself, which were already acceptable in the as-received conditions.

Further improvement of the procedure would include the use of a steel slab with hardened surface (e.g. after nitriding), in order to overcome the yield strength of the Ta sheet.

An alternative flattening procedure (not tested in this work) would consist in cold rolling a Ta sheet held between to flat steel plates, with the amount of passes required depending on the targeted final flatness.
